# Publisher’s Note: We Changed Page Numbers to Article Numbers for Articles Published in *Nursing Reports* Volumes 1–9

**DOI:** 10.3390/nursrep12010014

**Published:** 2022-02-28

**Authors:** Nursing Reports Editorial Office

**Affiliations:** MDPI AG, St. Alban-Anlage 66, 4052 Basel, Switzerland; nursrep@mdpi.com

*Nursing Reports* [1] was published by PAGEPress from Volume 1 (2011) to Volume 9 (2019). From Volume 10 (2020), *Nursing Reports* is published by MDPI.

Previous articles in Volumes 1–9, published by PAGEPress in Open Access under a CC-BY (or CC-BY-NC-ND) licence, are now hosted by MDPI on mdpi.com as a courtesy and upon agreement with PAGEPress.

To standardize the metadata format of all previous articles, MDPI republished 39 articles in Volumes 1–9 with article numbers replacing page numbers (Table 1).

## Figures and Tables

**Table 1:** MDPI changed page numbers to articles numbers for 39 articles in Volumes 1–9.

DOI	Previous Page Number	Current Article Number
10.4081/nursrep.2011.e1	1	e1
10.4081/nursrep.2011.e2	2–6	e2
10.4081/nursrep.2011.e3	7–14	e3
10.4081/nursrep.2011.e4	15–20	e4
10.4081/nursrep.2011.e5	21–23	e5
10.4081/nursrep.2011.e6	24	e6
10.4081/nursrep.2011.e7	25–28	e7
10.4081/nursrep.2011.e8	29–34	e8
10.4081/nursrep.2011.e9	35–39	e9
10.4081/nursrep.2012.e1	1–3	e1
10.4081/nursrep.2012.e2	4–12	e2
10.4081/nursrep.2012.e3	13–17	e3
10.4081/nursrep.2012.e4	18–24	e4
10.4081/nursrep.2012.e5	25–30	e5
10.4081/nursrep.2012.e6	31–38	e6
10.4081/nursrep.2012.e7	39–45	e7
10.4081/nursrep.2012.e8	46–51	e8
10.4081/nursrep.2012.e9	52–56	e9
10.4081/nursrep.2012.e10	57–62	e10
10.4081/nursrep.2012.e11	63–70	e11
10.4081/nursrep.2012.e12	71–79	e12
10.4081/nursrep.2013.e2	4–11	e2
10.4081/nursrep.2013.e3	12–17	e3
10.4081/nursrep.2013.e4	18–23	e4
10.4081/nursrep.2014.3225	1–6	3225
10.4081/nursrep.2014.3290	12–18	3290
10.4081/nursrep.2014.3647	7–11	3647
10.4081/nursrep.2015.4783	1–12	4783
10.4081/nursrep.2015.4849	29–31	4849
10.4081/nursrep.2015.5102	13–18	5102
10.4081/nursrep.2015.5134	22–23	5134
10.4081/nursrep.2015.5218	19–21	5218
10.4081/nursrep.2015.5306	24–28	5306
10.4081/nursrep.2016.5583	8–11	5583
10.4081/nursrep.2016.5665	1–7	5665
10.4081/nursrep.2017.6129	4–9	6129
10.4081/nursrep.2017.6155	1–3	6155
10.4081/nursrep.2018.6761	1–8	6761
10.4081/nursrep.2019.8041	1–5	8041
